# Advances in the Utilization of Zebrafish for Assessing and Understanding the Mechanisms of Nano-/Microparticles Toxicity in Water

**DOI:** 10.3390/toxics11040380

**Published:** 2023-04-17

**Authors:** Pengyu Lei, Wenxia Zhang, Jiahui Ma, Yuping Xia, Haiyang Yu, Jiao Du, Yimeng Fang, Lei Wang, Kun Zhang, Libo Jin, Da Sun, Junbo Zhong

**Affiliations:** 1 Institute of Life Sciences & Biomedical Collaborative Innovation Center of Zhejiang Province, Wenzhou University, Wenzhou 325035, China20160121@wzu.edu.cn (L.J.); 2 Department of Burn and Plastic Surgery, Zigong Fourth People’s Hospital, Zigong 643099, China; 3 Key Laboratory for Biorheological Science and Technology of Ministry of Education, State and Local Joint Engineering Laboratory for Vascular Implants, Bioengineering College of Chongqing University, Chongqing 400044, China

**Keywords:** zebrafish, nano-/microparticles, toxicity evaluation, toxicological mechanism

## Abstract

A large amount of nano-/microparticles (MNPs) are released into water, not only causing severe water pollution, but also negatively affecting organisms. Therefore, it is crucial to evaluate MNP toxicity and mechanisms in water. There is a significant degree of similarity between the genes, the central nervous system, the liver, the kidney, and the intestines of zebrafish and the human body. It has been shown that zebrafish are exceptionally suitable for evaluating the toxicity and action mechanisms of MNPs in water on reproduction, the central nervous system, and metabolism. Providing ideas and methods for studying MNP toxicity, this article discusses the toxicity and mechanisms of MNPs from zebrafish.

## 1. Introduction

**Figure 1:**
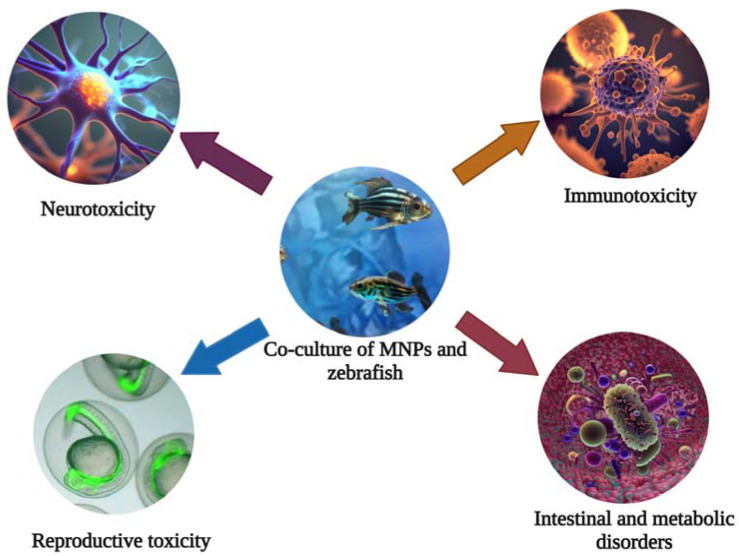
The zebrafish can be used for toxicity evaluation and mechanistic studies of the nervous, reproductive, and immune systems of MNPs.

Due to the continuous development of human activities, plastic products have become an integral part of our daily lives. However, with large quantities of plastic waste being discharged into water bodies, the global environment is expected to release 33 billion tons plastics of by 2050. In the course of environmental evolution, it will gradually decompose into tiny plastic particles called microplastics (MPs) (100–5000 nm) and nanoplastics (NPs) (<100 nm) [1,2]. Among the most common nano-/microparticles (MNPs) are polyethylene (PE), polypropylene (PP), polyvinyl chloride (PVC), polystyrene (PS), and polyethylene terephthalate (PET) [3]. Due to the small size, large surface area, strong adsorption capacity, and permeability of MNPs, their distribution and action mode in water are different from traditional organic pollutants [4,5,6,7]. High persistence and extreme distribution are unique characteristics of MNPs compared to other polluting suspended substances in the environment. As a result, MNPs are potentially harmful to freshwater and marine ecosystems [8,9]. Plastic debris has already caused an estimated EU 21 billion of severe economic damage in the world’s oceans. At the same time, due to their high surface area and hydrophobicity, MNPs can act as carriers of pathogens and organic pollutants, together with which they aggravate toxicity [10,11,12,13]. In addition, MNPs can enter the food chain circulation directly or indirectly, causing metabolic disorders and intestinal microbiota disorders in the human body. In addition, they cause neurotoxicity, reproductive toxicity, immunotoxicity, etc., which constitute serious threats to human health (Figure 1) [11,14,15]. 

Therefore, to assess the toxicity of MNPs in water, many toxicity evaluation methods have been developed, including *in vitro* and *in vivo* methods. *In vitro* tests include chemical analysis and cytotoxicity tests [16,17]. These methods have limitations such as not reflecting real environmental conditions and complicated operation, and are time-consuming and laborious. A variety of animal models have been used to evaluate MNP toxicity *in vivo*, including mice, rats, rabbits, guinea pigs, and fish [18,19,20,21]. The zebrafish is a small freshwater fish. (I) Because of its small size, vitality, and reproductive ability, it is easy to raise and manage [22]; (II) its short life cycle, from fertilized egg to adult in about three months, allows for rapid large-scale experiments [23]; (III) Zebrafish larvae are transparent, and the development process of their internal structures and organs can be directly observed through a microscope. Therefore, they emerge in the field of toxicity evaluation of pollutants such as MNPs, heavy metals, pharmaceuticals, and algae in water [24].

This review summarizes the toxicity evaluation and mechanism research of the nervous, reproductive, and immune systems of the zebrafish used in MNPs, pointing out the challenges faced by the zebrafish in the toxicity evaluation of MNPs, and suggesting improved solutions, in order to facilitate better application of the zebrafish to toxicity evaluations of MNPs and exploration of their mechanisms of action.

## 2. Reliability of the Zebrafish for Toxicity Evaluation

**Figure 2:**
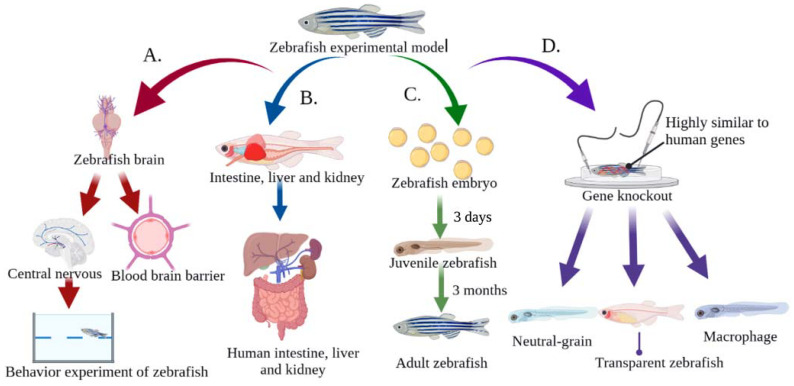
Advantages of zebrafish for toxicity evaluation of MNPs. (**A**) The blood–brain barrier, central nervous system, and social behavior are similar to those of humans, making it an ideal animal model for studying neurotoxicity; (**B**) The intestine, liver and kidney are similar to those of humans, which makes them suitable for studying MNPs metabolism and immune diseases; (**C**) The reproductive and developmental toxicity of MNPs can be easily studied due to the short reproductive cycle of zebrafish and the large number of eggs laid. In addition, the embryos are transparent, so a microscope can be used to observe the cell division and organ formation process; (**D**) The genome of zebrafish has been fully sequenced, and is highly consistent with the human genome, and can be easily manipulated by genetic manipulation such as gene knockout and gene overexpression.

Traditionally, mammals have been used to assess toxicity. Although the mammalian model has many advantages, it also has a number of disadvantages, such as its high cost, lengthy process time, and ethical and moral issues. Conversely, zebrafish are known for their fast population growth, short reproductive cycle, ease of experimental operation, high survival rate, and low feeding and maintenance costs [25,26]. Figure 2 illustrates the main advantages of using zebrafish in MNP research MNPs. These advantages include: (I) the genome of zebrafish has been completely sequenced, and the gene similarity to humans is as high as 87%. In addition, the pathological state of many diseases and genes related to disease etiology are highly conserved in humans [27,28]; (II) The transparent nature of zebrafish embryos and larvae provides an experimental advantage compared to other model organisms for studying the accumulation sites of fluorescence-labeled MNPs particles. In addition, transgenic zebrafish strains have become effective biological models for studying metabolic and immune diseases [29,30]; (III) The blood–brain barrier of zebrafish is similar to that of humans, and has been well used for central-nervous-system drug screening [31]; (IV) The nervous system of the zebrafish, which includes the central nervous system and the peripheral nervous system, has social behaviors similar to human perception, movement, and emotion. Zebrafish have become widely used models in behavioral neuroscience, especially as disease models for Parkinson’s disease (PD), Alzheimer’s disease (AD), and depression [32,33,34]; (V) Zebrafish have similar metabolic organs and physiological structures to humans, such as the liver, kidney, and intestine. These organs can metabolize and eliminate harmful substances as shown in Figure 2 [35,36,37].

Zebrafish are also being used in toxicity assessment, according to many studies. Zebrafish have been shown to exhibit physiological responses and behavioral abnormalities as a result of exposure to environmental pollutants that are similar to those of mammals. As a result, zebrafish have been widely utilized in toxicity assessments, drug screening, and other fields, demonstrating their reliability for evaluating the biological effects of MNPs.

## 3. Toxicity of MNPs in Water to Zebrafish 

**Figure 3:**
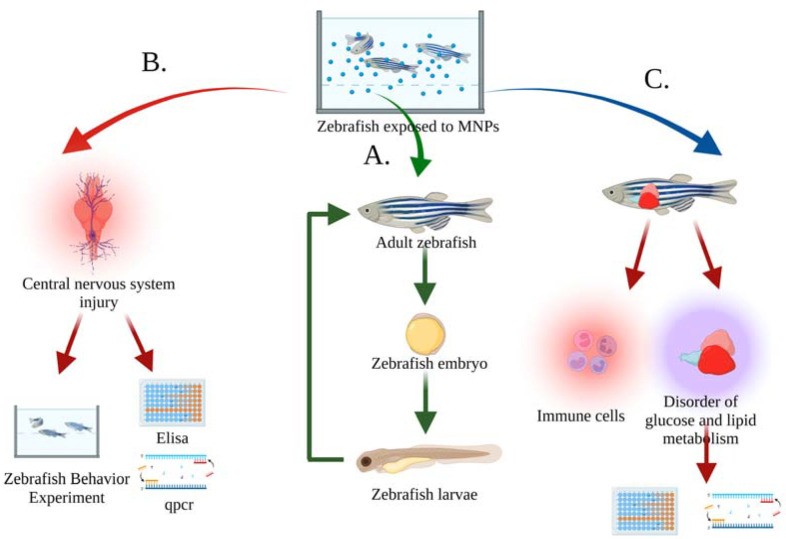
Evaluation index of MNP toxicity in zebrafish in a water body. (**A**) Evaluation of the toxicity of MNPs on growth and reproduction by the damage and apoptosis of sperm, testis, and oocytes of adult zebrafish, as well as the increased rate of malformation and mortality of embryos [40,41,42]; (**B**) Evaluation of the behavioral and neurological toxicity of MNPs by increasing oxidative-stress level and apoptosis in the zebrafish brain, as well as behavioral experiments to detect memory, learning, and mental disorders in zebrafish [43,44,45]; (**C**) Evaluation of the toxicity of MNPs on the metabolism and immune system by the upregulation of immune-related gene expression and apoptosis, as well as the reduction of hepatic glucose and lipid metabolism, glucose, α-ketoglutarate, and lipid-related indicators [46,47,48].

Currently, the zebrafish used to evaluate MNP biotoxicity are manipulated by the exposure method [38,39], injecting MNPs directly into the aquaculture water or adding MNPs to the feed, and then exposing the zebrafish to the suspension for a specified period of time. Zebrafish behavior and physiological indicators can be observed during this period. As shown in Figure 3, acute toxicity of MNPs to zebrafish was evaluated by observing changes in growth, reproduction, behavior, and physiology. In addition, water-flow can also control MNP concentration and time to simulate dynamic MNP exposure environments.

### 3.1. Growth and Reproduction of Zebrafish

Exposure of zebrafish to MNPs in aqueous solution has adverse effects on the growth and reproduction of zebrafish: (I) It may cause damage to the chorionic membrane of zebrafish embryos or changes in water quality (such as hypoxia induction) [41], which may lead to premature hatching of embryos, and their inability to survive for a long time, resulting in an increase in the mortality of larvae [49]; (II) Zebrafish larvae were unable to survive for long in an aqueous solution containing 2 mg/mL MNPs, and their mortality rate increased to 32.4%; (III) Exposed larvae displayed edema of the yolk sac and pericardium, curvature of the spine, curvature of the tail, and a larger area of visual vesicles; (IV) When zebrafish embryos were exposed to 100 ppm of PET-NPs in aqueous solution, the survival rate decreased to 65%, while at 200 ppm the survival rate was almost zero [50]; (V) A previous study exposed adult zebrafish to PS-NPs for one month, and a large accumulation of PS-NPs, as well as an increase in reactive oxygen species (ROS) was observed in the gonads and liver of the zebrafish [51]. It was found that high concentrations of MNPs resulted in apoptosis of male zebrafish testis, as well as a significant reduction in basement-membrane thickness, resulting in oxidative stress to female zebrafish oocytes, leading to apoptosis [40], and affecting egg morphology and yolk area of zebrafish offspring, resulting in malformations [52]; (VI) Upon exposure to PE-MPs in water for 15 days, adult zebrafish accumulated a large amount of PE-MP in their bodies, causing DNA damage to red blood cells and nuclear abnormalities, which may lead to genetic damage. In addition, the activities of superoxide dismutase (SOD) and catalase (CAT) decreased, resulting in an imbalance in the redox state [53].

### 3.2. The Behavior and Nervous System of Zebrafish

According to studies, MNPs can cross the blood–brain barrier [54], and they accumulate in large amounts in the heads of zebrafish, causing an increase in oxidative stress levels and apoptosis in the brain [43,44], in addition to a decrease in the activity of acetylcholinesterase (AChE) in the brain of the zebrafish (the activity regulates brain function and is considered a biomarker of neurotoxicity in zebrafish), as well as inhibition of the synthesis of neurotransmitters (such as dopamine, melatonin, aminobutyric acid, and 5-hydroxytryptamine) [55,56], causing serious harm to the nervous system and inducing neurobehavioral disorders [57]. One study found that zebrafish swimming behavior and range of motion were significantly reduced when exposed to PC-MPs and PE-MPs in water. There were also emetic and electrophysiology abnormalities, learning and memory problems [58,59], and severe epileptic behaviors [45]. Meanwhile, avoidance and anxiety behaviors were observed in zebrafish larvae [60]. The above studies suggest that MNP exposure may negatively affect the nervous system and behavior of zebrafish. This provides a rapid method for assessing MNP toxic effects on aquatic organisms’ nervous systems and behavior.

### 3.3. Metabolism and Immune System of Zebrafish

MNPs are capable of crossing biological barriers and entering the circulatory system, where they reduce energy reserves through mechanical destruction, ultimately affecting the immune system [61]. (I) It was found that zebrafish exposed to MNPs had significant increases in mRNA and protein levels of genes associated with the innate immune system (interleukin-1α (IL-1α), IL-1β, nuclear factor-κb (NF-κb), and interferon) and apoptosis (casp3A and BCL2) [62]. Moreover, the number of white blood cells decreased, the accumulation of neutrophils in intestinal epithelium increased, and the immune function was abnormal [63]. At the same time, the metabolic system of zebrafish was adversely affected, resulting in abnormal liver and intestinal metabolism; (II) Zebrafish embryos exposed to PE-MPs showed an interference in the metabolism of triglycerides, total cholesterol, non-esterified fatty acids, total bile acids, glucose, and pyruvate; (III) When adult male zebrafish were exposed for 21 days to PS-MPs, a significant reduction in body weight was observed as well as a reduction in transcription levels associated with glucose and lipid metabolism in the liver. Furthermore, glucose, pyruvate, and alpha-ketoglutaric acid levels in the liver decreased. The liver also decreased in genes related to fatty-acid metabolism and amino-acid metabolism; (IV) In adult zebrafish exposed for 21 days to aqueous solutions of PE-MPs and PP-MPs, MPs induced oxidative stress, resulting in lipid peroxidation as well as stimulation of autophagy and apoptosis signal transduction pathways [64,65]. In combination, exposure to MNPs disrupts the immune system and glucose and lipid metabolism in zebrafish (Table 1) [46].

## 4. Exploring the Toxicological Mechanisms of MNPs in Water with Zebrafish

**Figure 4:**
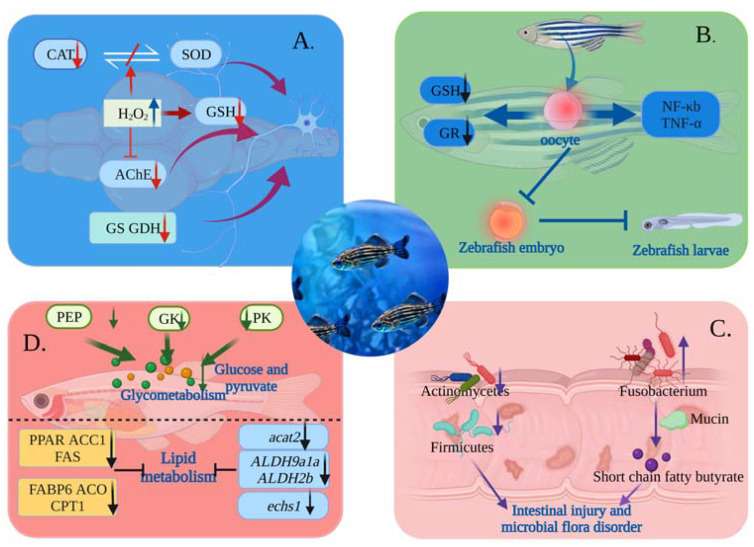
Diagram of the toxicological mechanism of MNPs in water explored using zebrafish. (**A**) The mechanism of central nervous toxicity of MNPs: Excessive production of H_2_O_2_, inactivation of enzyme protein, disruption of the balance between CAT and SOD activities, inhibition of AChE activity, and significant reduction in the activity of GS and GDH, leading to brain neurodegeneration. (**B**) Mechanism of reproductive toxicity of MNPs: MNPs were transferred to oocytes of adult female zebrafish, causing reduction of GR and GSH contents, leading to oxidative stress in oocytes. At the same time, NF-b and TNF- expression was significantly upregulated, affecting the offspring’s growth and development. (**C**) Effect of MNPs on zebrafish gut: Increased abundance of Fusobacterium metabolized mucin into short-chain fatty butyrate and induce intestinal inflammation in zebrafish. The number of actinobacteria decreased, foreign bodies accumulated in the intestine, and the number of Firmicutes decreased, all of which resulted in reduced mucus production and damage to the intestinal barrier. (**D**) The mechanism of metabolic toxicity of MNPs: Reduction of PEP, GK, and PK in the glycolytic pathway led to insufficient glucose and pyruvate production, leading to disorders of glucose metabolism. Additionally, the expression levels of *PPAR*, *ACC1*, *FAS*, *FABP6*, *ACO*, *CPT1*, *acat2*, *ALDH9a1a*, *ALDH2b*, and *echs1* genes were reduced, resulting in abnormal lipid metabolism.

The genes, blood–brain barrier, and central nervous system of zebrafish are highly similar to those of humans, and at the same time, the zebrafish have the advantage of rapid reproduction, more eggs, zebrafish larvae transparent intestines, etc. Therefore, it is easy for researchers to examine how MNPs affect the nervous system, intestinal microorganisms, reproductive development, and immune system toxic mechanisms (Figure 4).

### 4.1. Toxicological Mechanism of MNPs on Nervous System

Adult zebrafish were exposed for a long period of time to a PS-MNP solution in an attempt to detect the effects of MNPs on the oxidative stress system, the cholinergic system, the glutaminergic system, and the histological changes in the brain [44]. By reducing CAT activity, a key antioxidant defense mechanism, and glutathione (GSH) levels, PS-MPs induce oxidative stress. Overproduction of H_2_O_2_ inactivates enzyme proteins and disrupts CAT and SOD activity balance. GSH is also a powerful antioxidant, protecting cells against oxidative damage by removing ROS and pro-oxidants. It also plays an essential role in maintaining cell redox homeostasis. Cell detoxification requires GSH combined with MNPs. Additionally, excess H_2_O_2_ will cause glutathione to be oxidized into glutathione disulfide in the glutathione redox cycle [69], which contributes to oxidative stress reactions [70,71,72].

At the same time, AChE activity decreases and excess acetylcholine neurotransmitter (ACh) in synapses leads to interruption of normal nervous-system function, which can cause some neurodegenerative diseases (AD, dementia with Lewy bodies, PD, etc.) [73,74,75]. In zebrafish soaked in MNPs, abnormal neurobehavioral phenomena were observed which may have been the result of oxidative stress caused by the damage of the endoplasmic reticulum, Golgi apparatus, and cell membrane, which may have led to blocking of the AChE enzyme production [76]. In addition, H_2_O_2_ and AChE metabolism are directly correlated. As an allosteric activator of AChE, H_2_O_2_ can change the structure and distribution of isomers in cells. It has an antagonistic effect on AChE activity [77].

The glutaminergic system is the main excitatory neurotransmitter system in the brain. Glutamate is considered the most abundant neurotransmitter in the central nervous system. It is crucial in many aspects of central-nervous-system function, including cognition, memory, and learning. Glutamine synthetase (GS) and glutamate dehydrogenase (GDH) are two major enzymes involved in brain glutamate metabolism. GS maintains the balance of the glutamine–glutamine and ammonia cycles. Decreased GS activity leads to increased glutamate and ammonia concentrations. In the presence of excessive amounts of extracellular glutamate, glutamate receptors are overactivated, resulting in neuronal damage and cell death [78]. Studies have shown that MNPs immersed in the solution will transfer to the brain of zebrafish. They will interfere with glutamate metabolism, and significantly reduce GS and GDH, which may cause brain neurodegeneration. In addition, histological analysis of neuronal filament populations and myelin axon sites in the white layer of the brain showed that MNPs caused severe degradation of the brain-cell layer. This significantly reduced the number of neurons and nerve cells [44].

In conclusion, MNPs can cause central neurotoxic effects by negatively affecting various biomarkers in the central nervous system of zebrafish by negatively affecting various biomarkers. In doing so, CAT activity and GSH levels are reduced, resulting in oxidative damage, interfering with glutamatergic and cholinergic neurotransmitter systems, and resulting in brain cell apoptosis. Thus, zebrafish are a compelling choice as a model organism for neurotoxicity studies.

### 4.2. Toxicological Mechanism of MNPs Affecting Reproduction

The effects of exposure to PS-NPs on adult zebrafish (F0) and the effects of PS-NPs on zebrafish physiology have been investigated. The experiment produced four F1 groups: blank control, exposed female, exposed male, and exposed male and female. Detection of F1 embryos and larvae showed that NPs were present in yolk sacs, gastrointestinal tract, liver, and pancreas of female and male F1 embryos, and larvae exposure to NPs, possibly due to the high affinity of NPs for plasma proteins (lipid transport proteins, vitellinogen, and zona pellucinalis) [79] and its interaction with vitellinogen, promoted the transfer of PS-NPs to oocytes and eventually to the yolk sac of the embryo; thus, PS-NPs were transferred from females to offspring [80]. Furthermore, bradycardia was observed only in females and both male and female injected embryos. This may be associated with PS-NPs entering the cells and interacting with the myocardium of the heart, thereby altering cardiac function [40].

It was also found that glutathione reductase (GR) activity and mercaptan levels were reduced in larval brains, muscles, and testes. GR may have an important role to play in early development. Its decreased activity may lead to long-term physiological effects after maturity and increase the sensitivity of organisms to other environmental stressors. It is also possible that the reduction in mercaptan levels is due to the consumption of mercaptan (such as GSH) during PS-NPs detoxification. Therefore, PS-NPs will increase offspring susceptibility to oxidative stress. In conclusion, PS-NPs may bioaccumulate and be passed on to offspring, altering the antioxidant system, but do not cause significant physiological damage [41].

Meanwhile, isolated zebrafish oocytes were incubated for 6 h with different doses of PS-NPs and the effects of PS-NPs on the zebrafish oocytes were quantified by real-time PCR. PS-NPs induced oxidative stress in oocytes and increased ROS production in the cells, affecting immune regulatory genes that play a critical role in oocyte maturation. Therefore, PS-NPs can inhibit DNA repair and apoptosis pathways, causing cell death. In addition, they led to significant upregulation of pro-inflammatory response genes (NF-κb and tumor necrosis factor-α (TNF-α)), indicating disruption of the normal ovulation process; therefore, PS-NPs impair reproductive function in zebrafish.

### 4.3. Effect of MNPs on Gut Histopathology and Microbiota in Zebrafish

The toxicological effects of MNPs are closely related to their size [81]. Since NPs have a large specific surface area and complex surface structure, their retention and purification time in the organism are longer, and their bioaccumulation rate is higher than MPs. Following MNP uptake in zebrafish, the gut is the main site of accumulation [82]. Zebrafish were exposed to PS-MNPs of different sizes for 30 days, which resulted in a change in intestinal tissues and microbial fractions. MNPs damaged gastrointestinal histology size-dependently. Smaller MNPs accumulated more in the intestine [83,84]. According to histological analysis, the intestinal tissue was severely damaged by necrosis and exfoliation of the top of the intestinal villi. This was followed by abrasion of crypt cells, structural tissue lysis, and vacuolization of intestinal epithelial cells [85].

Furthermore, *Fusobacteria* numbers increased, *Firmicutes* and *Actinobacteria* numbers decreased slightly, and the abundance and diversity of intestinal microbes decreased significantly. The increase in *Fusobacteria* could cause intestinal injury. *Fusobacteria* metabolize carbohydrates (including mucin) into short-chain fatty butyrate, which causes intestinal inflammation. There is even a cancer risk [86,87]. Additionally, actinomycetes have gained worldwide attention for their ability to synthesize secondary metabolites that remove foreign bodies. Therefore, a reduction in actinomycetes may explain MNP damage [88]. In addition, some *Firmicutes* produce butyrate, which provides nourishment and energy to epithelial and intestinal cells. It also increases mucus production and decreases inflammation, which may impair the integrity of the intestinal barrier if *Firmicutes* are reduced in abundance [89,90,91]. To conclude, long-term exposure to MPs may lead to intestinal flora disorders, inflammation of the intestinal tract, and cytokines that influence immunity. It may also trigger a series of immune responses and produce oxidative stress, which could threaten the health of the zebrafish’s microbiota and immune system.

### 4.4. Toxicological Mechanisms of MNPs Causing Metabolic Disorders

By exposing adult zebrafish to PS-MPs for 21 days, it was shown that PS-MPs had hepatic effects related to glucose and lipid metabolism [48]. Results indicated that PS-MPs may cause liver metabolic disorders in zebrafish by blocking the digestive tract and inhibiting enzyme production [92,93]. In addition, the liver is primarily responsible for bioaccumulation, metabolism, and detoxification. Therefore, analysis of the major genes involved in glucose and lipid metabolism in zebrafish liver showed that the expression levels of rate-limiting enzyme genes such as cytoplasmic phosphoenolpyruvate carboxykinase (PEP), glucokinase (GK), and pyruvate kinase (PK) in the glycolytic pathway were reduced. Therefore, glucose and pyruvate production were insufficient, resulting in glucose metabolism disorders [94,95,96]. At the same time, alterations in several genes that regulate hepatic lipogenesis, for example, decreased expression of peroxisome proliferator-activated receptor (PPAR) family genes, acetyl-coa carboxylase 1 (ACC1), and fatty acid synthase (FAS) genes, have been associated with downregulation of triglyceride (TG) [97]. In addition, the reduction of α-ketoglutarate and isocitrate dehydrogenase (ICD), which are involved in tricarboxylic acid cycle energy metabolism, implies that MP exposure reduced energy metabolism in zebrafish. Furthermore, this study showed that the expression of fatty acid binding protein 6 (FABP6), which regulates lipid transport and acyl-coa oxidase (ACO), which participates in lipid oxidation, and carnitine palmitoyltransferase 1 (CPT1), was reduced [46,98].

In addition, reduced gene expression levels of *acat2* (which protects hepatocytes from excess cholesterol [99]), as well as of *ALDH9a1a* and *ALDH2b* (aldehydes are irreversibly converted into acids in the liver, and large increases in aldehydes may result in enzyme inactivation and DNA damage, among other things [100,101]), and *echs1* (enoyl-coa hydratase 1 is an important gene in fatty acid metabolism [102]) further confirmed that MP exposure can affect glucose, lipid, and amino acid metabolism. In addition, it provides substantial information regarding MPS-induced metabolic disorders in aquatic animals [47].

The zebrafish has provided a preliminary understanding of MNP toxic mechanisms in the nervous system, gut microbiota, reproductive development, and immune system. Furthermore, it provides an excellent animal model for future studies, treatment, and attenuation of MNPs toxic effects.

## 5. Limitations and Possible Solutions

**Figure 5:**
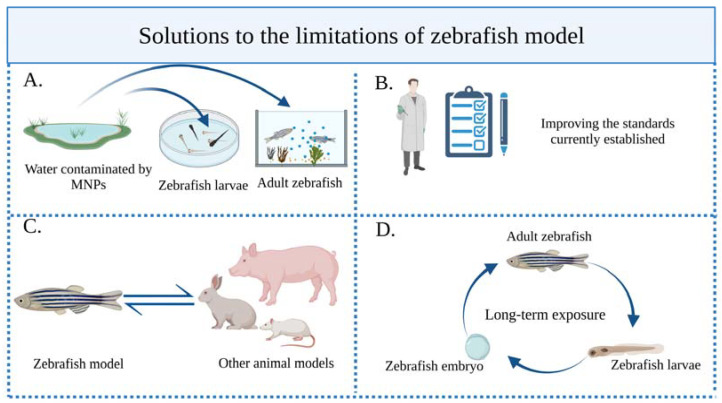
Solutions to the limitations of zebrafish. (**A**) To optimize the zebrafish exposure experiments, larval or adult zebrafish should be exposed to real MNPs in contaminated water. (**B**) The current unified experimental operating procedures and standards must be strictly followed, and researchers still need to promote the construction of standards, so that the toxicity evaluation of zebrafish as a model organism can be more perfect. (**C**) The mutual verification mechanism for animal models must be strengthened. (**D**) Long-term exposure experiments should be carried out to expose adult zebrafish to MNPs over a long period of time and there should be generation studies from adult fish to offspring, and then to offspring adult respawning to comprehensively investigate MNP toxic mechanisms.

MNPs have complex effects and mechanisms, which require accurate, sensitive, and repeatable methods to evaluate. As an animal model for toxicity assessment, zebrafish are widely used in MNP toxicity assessment. Despite its many advantages in assessing MNP toxicity in water, the zebrafish is used as a non-mammal model. There may be some problems with toxicity assessment results, such as species differences, repeatability, and a lack of long-term toxicity research. It is imperative to take these limitations into account when assessing MNP toxicity with the zebrafish.

(I) MNP particles have a very small size and large surface area, making their behavior and effects in water complex, so it is difficult to accurately simulate the real situation. To simulate the real situation in water, further study and optimization of the zebrafish exposure experiment design and method is necessary.

(II) The experimental results of different laboratories may differ due to zebrafish physiological characteristics and reproduction mode. To improve reproducibility and comparability of experiments, it is necessary to strictly follow the currently developed uniform experimental operating procedures and standards (as FET test) [103]. However, researchers still need to continuously promote the construction of standards to make zebrafish, as a model organism for toxicity evaluation toxicity, more perfect. In addition, for persuasive purposes, genetically consistent or inbred animal strains should be used.

(III) Lack of long-term toxicity studies: Most studies are short-term exposure experiments, with limited long-term data available. The reason for this is that zebrafish exposure experiments typically use high concentrations of compounds. By doing so, toxic effects of low concentrations of compounds can be evaluated rapidly. However, they cannot assess the potential toxic effects of low concentrations. A long-term exposure test can be used to better simulate the actual situation and explore MNPs’ long-term toxic effects. To enhance persuasion, more long-term exposure experiments are needed.

(IV) Zebrafish cannot replace the mammalian model completely: Although zebrafish have many advantages, their physiological characteristics and living environment are fundamentally different from those of humans. In practical applications, it is therefore necessary to establish a mutual verification mechanism between zebrafish and various animal models and combination methods. This will enable us to conduct a more comprehensive assessment of the biological toxicity of MNPs (Figure 5). 

In addition, computer simulations and molecular biology technologies can also be used to study MNP toxicity mechanism and action targets. This provides a theoretical basis for more accurate and rapid evaluation methods.

## 6. Conclusions

Water pollution caused by MNPs has become a global environmental issue. The zebrafish has emerged as a promising animal model to understand the toxic impacts and mechanisms of MNPs in water. It provides a novel solution for mitigating MNP pollution. The focus of future research will be on zebrafish research and optimization, and will incorporate the rapid evaluation of several parameters of different indicators, as well as gene-editing technology for the construction of zebrafish mutation models, including genetic and psychiatric disease models, so that zebrafish research can be conducted more efficiently, accurately, and reliably. Furthermore, it is imperative to bolster fundamental research on MNP pollution and better understand its physical and chemical characteristics, environmental behavior, and biological mechanisms. This will provide a more comprehensive and accurate theoretical foundation for zebrafish toxicity evaluations.

## Figures and Tables

**Table 1:** Summary table of toxicity caused by different MNPs in zebrafish.

Toxicity of MNPs	Types of MNPs	Experimental Model	Days of Exposure	Harm Caused	References
Growth and reproductive toxicity	PS-NPs	Adult zebrafish and their offspring	Adult zebrafish: 7 days, larvae: 96 h	Significantly reduced glutathione reductase activity in adult zebrafish brain, muscle, and testis. In addition, GSH reductase activity and thiol levels were decreased and the oxidative system was disrupted in embryos and larvae of female and co-parent exposed offspring.	[41]
	PET-NPs	Zebrafish embryos and larvae	96 h	Embryo hatching rate and survival rate decreased dramatically, accompanied by impaired mitochondrial-membrane integrity and significant alterations in oxidative-stress-related pathways.	[50]
	PE-NPs	Adult zebrafish	360 days	Zebrafish fecundity, egg morphology, and yolk area were impaired and skeletal deformities and impaired development of the caudal fins and scales. Malformations were also observed in the offspring.	[52]
	PVC-MPs	Zebrafish embryos and larvae	9 days	It delayed embryo hatching, cause zebrafish death and induce oxidative stress, and inhibited heart development.	[66]
	PE-MPs	Adult zebrafish	15 days	It caused DNA damage and nuclear abnormality in red blood cells, as well as a decrease in SOD and CAT activities, and a large amount of ROS was produced, which caused oxidative stress.	[53]
	PS-NPs	Zebrafish oocytes	6 h	It caused oxidative stress, immunotoxicity, and apoptosis in oocytes.	[40]
Neurotoxicity	PS-MPs	Adult zebrafish	7 days	Zebrafish exhibited anxious behavior, increased swimming distance, and longer periods of manic and active states.	[58]
	PS-NPs	Adult zebrafish	7 days	PS-NPs accumulated in zebrafish brains and the fish exhibited circadian rhythm activity, aggressive behavior, anxiety behavior, and predator-avoidance behavior. In addition, the expression of neurotoxicity-related neurotransmitters such as dopamine, 5-hydroxytryptamine, and γ-aminobutyric acid was decreased.	[51]
	PE-MPs	Adult zebrafish	4 days	The zebrafish showed erratic movements and epileptic behavior with the tail bent downward or upward	[45]
	Mixture of MPs	Zebrafish larvae	14 days	It reduced the average swimming speed and total distance of zebrafish larvae. It affected avoidance behavior and aversive stimulus response, and significantly inhibited the AChE activity of zebrafish larvae.	[60]
	PS-NPs	Zebrafish larvae	120 h	Comparing PS-NH_2_ (positively charged) to PS-COOH (negatively charged), PS-NH_2_ caused greater developmental toxicity and apoptosis in the brain and greater neurobehavioral impairment as well as a reduction in glycine, cysteine, glutathione, and glutamate levels.	[43]
	PS-NPs	Zebrafish embryos and larvae	120 h	PS-NPs entered the embryo and brain, harmed embryonic development, induced neuronal loss, specifically interfered with the GABAergic, cholinergic, and serotonergic systems, and affected neuronal signaling, resulting in behavioral abnormalities.	[67]
Metabolic and immune system toxicity	PS-MPs	Zebrafish larvae	8 days	The expression of genes related to oxidative stress and immune system response was upregulated, and cell apoptosis occurred.	[62]
	PE-MPs	Zebrafish embryos and larvae	7 days	Glucose and lipid metabolism was disturbed, and the metabolism of triglycerides, total cholesterol, non-esterified fatty acids, total bile acids, glucose, and pyruvate was disturbed.	[46]
	PS-MPs	Adult male zebrafish	21 days	Glucose, lipid, and amino-acid metabolism in the liver was disordered, and the amounts of glucose, pyruvate, and α-ketoglutarate in the liver were decreased.	[48]
	PE-MPs	Adult zebrafish	21 days	It caused DNA damage and increased ubiquitination levels, while causing oxidative stress, leading to lipid peroxidation and apoptosis.	[65]
	PP-MPs	Adult zebrafish	21 days	It caused lipid peroxidation, DNA damage, protein ubiquitination, cell apoptosis, and autophagy, and inhibited the functions of zebrafish gill and liver cells.	[64]
	PS-MPs	Adult zebrafish	7 days	It induced oxidative stress and disrupted fat and energy metabolism, resulting in liver inflammation and lipid accumulation in zebrafish.	[68]

## Data Availability

Not applicable.

## Abbreviations

MNPs	Nano-/microplastics
MPs	Microplastics
NPs	Nanoplastics
PE	Polyethylene
PS	Polystyrene
PP	Polypropylene
PVC	Polyvinyl chloride
PET	Polyethylene terephthalate
PD	Parkinson’s syndrome
AD	Alzheimer’s disease
ROS	Reactive oxygen species
AChE	Activity of acetylcholinesterase
IL-1α	Interleukin-1α
NF-κb	Nuclear factor-κb
CAT	Catalase
GSH	Glutathione
SOD	Superoxide dismutase
ACh	Acetylcholine neurotransmitter
GS	Glutamine synthetase
GDH	Glutamate dehydrogenase
GR	Glutathione reductase
TNF-α	Tumor necrosis factor-α
PEP	Phosphoenolpyruvate carboxykinase
GK	Glucokinase
PK	Pyruvate kinase
PPAR	Peroxisome proliferator-activated receptor
ACC1	Acetyl-coa carboxylase 1
FAS	Fatty acid synthase
ICD	Isocitrate dehydrogenase
FABP6	Fatty acid binding protein 6
ACO	Acyl-coa oxidase
CPT1	Carnitine palmitoyltransferase 1
Echs1	Enoyl-coa hydratase 1
TG	Triglyceride
